# Allergic diseases of the skin and drug allergies – 2022. Consensus statement on management of urticaria in India

**DOI:** 10.1186/1939-4551-6-S1-P109

**Published:** 2013-04-23

**Authors:** Kiran Godse

**Affiliations:** 1 Dermatology, DR.D.Y.Patil Medical College and Hospital, India

## Background

Urticaria, a heterogeneous group of diseases, often cannot be recognized by its morphology. Due to non-specific and non-affordable diagnosis, management of urticaria, especially chronic urticaria, is very challenging. This guideline includes definition, causes, classification and management of urticaria. Urticaria has a profound impact on the quality of life and causes immense distress to patients, necessitating effective treatment. One approach to manage urticaria is identification and elimination of the underlying cause(s) and/or eliciting trigger(s), while the second one is treatment aimed at providing symptomatic relief. This guideline recommends use of second-generation non-sedating H1 antihistamines as the first-line treatment. The dose can be increased up to four times to meet the expected results. In case patients still do not respond, appropriate treatment options can be selected depending on the cost.

First-generation antihistamines can interfere with rapid eye movement (REM) sleep and impact on learning and performance. New GA²LEN/EDF/EAACI/WAO guidelines do not recommend the use of these sedating antihistamines for the routine management of CU as the first-line agents. Second-generation antihistamines should be considered as the first-line symptomatic treatment for urticaria because of their good safety and tolerability profile.Second-generation antihistamines in higher doses have been shown to be effective in control of chronic spontaneous urticaria. This has been verified in studies using even up to fourfold higher than recommended doses of desloratadine, fexofenadine, levocetirizineand rupatadine.

## Methods

Approach to chronic spontaneous urticaria.

## Results

**Figure 1:**
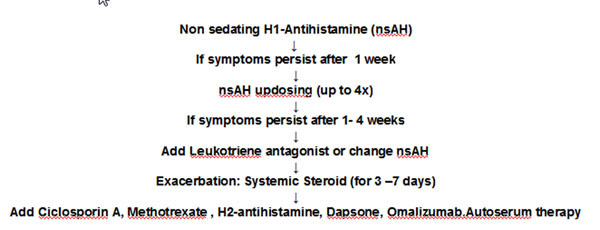
**Results Diagram**.

See Results Diagram in Figure 1.

## Conclusions

Four-fold updosing of antihistamines is recommended in urticaria management by Indian consensus statement.

